# A clinical study of inferior alveolar nerve damage caused by Carnoy’s solution used as a complementary therapeutic agent in a cystic lesion

**DOI:** 10.1186/s40902-020-00257-4

**Published:** 2020-05-25

**Authors:** Hyun-Jun Jo, Hee-Youl Kim, Dong-Cheol Kang, Dae-Ho Leem, Jin-A Baek, Seung-O Ko

**Affiliations:** Department of Oral and Maxillofacial Surgery, School of Dentistry, Chonbuk National University Dental Hospital, 20, Geonji-ro, Deokjin-gu, Jeonju-si, Jeollabuk-do Republic of Korea

**Keywords:** Carnoy’s solution, Hypoesthesia, Paresthesia, Sensory dysfunction, Inferior alveolar nerve damage

## Abstract

**Background:**

Cyst enucleation, which extracts only the tumor with the application of Carnoy’s solution (CS), has been suggested as a conservative treatment with a low recurrence rate and morbidity. However, there has been a concern that CS’s contact with inferior alveolar nerve (IAN) can cause neurons to degenerate and cause sensory dysfunction. The purpose of this retrospective cohort study aimed to investigate the neurosensory function after surgical treatment with or without the application of CS.

**Methods:**

While controlling the effects of sex, age, follow-up period, and invasion size of the tumor, we performed the binary logistic regression analysis to examine whether or not the sensory function of the patients who were treated with CS (*n* = 19) for the cyst enucleation procedure was significantly different from those who were not treated with CS (*n* = 58) at the end of the follow-up period.

**Results:**

The logistic regression result showed that the use of CS was not significantly related to the normalness of sensory function at the end of the follow-up period. Rather, the invasion size of the cyst was significantly associated with sensory dysfunction.

**Conclusions:**

CS may be used for patients who are diagnosed with OKC and UAM without much fear of its impact on sensory dysfunction. However, a small number of patients who were treated with CS experienced severe sensory damage and did not recover at the end of the follow-up period, suggesting the need for further analysis of these patients.

## Background

Carnoy’s solution (CS) is a chemically cauterizing agent that has been used to treat various cysts and tumors of aggressive nature in the oral and maxillofacial surgery area. Although many studies indicated that the use of CS is effective to reduce the recurrence of various cysts and tumors in the oral and maxillofacial surgery area, such as odontogenic keratocyst (OKC), unicystic ameloblastoma (UAM) [1–4], researchers have not examined the effect of CS on the inferior alveolar nerve (IAN) and sensory dysfunction. Therefore, it is important to research the relationship between the use of CS and sensory dysfunction.

In particular, CS has been commonly used for the treatment of odontogenic keratocyst (OKC) and unicystic ameloblastoma (UAM) and one of the three clinical variants of ameloblastoma (AM). CS composed of 60 ml (1 mol: 46.07 g) of absolute alcohol (ethanol), 30 ml (1 mol: 119.38 g) of chloroform, and 10 ml (1 mol: 60.05 g) of acetic acid. The penetration of this solution into tissues results in rapid local fixation and denaturation [5]. CS has been reported to have systemic toxicity that can cause local damage to anatomical structures, especially nerves [6]. However, Blanas et al. [7] suggested that there would be no damage to the nerves if the inferior alveolar nerve (IAN) is not exposed to CS for more than 3 min [7].

The inferior alveolar nerve (IAN) is located on the mandible. If the sensory nerves are damaged, the hypoesthesia or paresthesia may occur. Therefore, IAN is a structure that should be preserved as much as possible without much damage [8, 9]. It is reasonable for an oral surgeon to choose cyst enucleation with CS rather than extensive resection to preserve IAN and the surrounding bone tissue when the lesion contains IAN. The surgeon needs to remove the lesion carefully while protecting the nerves. At this time, CS can be applied with chemical cauterization ability because OKC, UAM daughter’s cyst, and epithelial cell residue exist in the space adjacent to the nerve of the bone and cause recurrence. However, CS may damage neurons beyond the peripheral sheath of the nerve and cause sensory dysfunction when CS comes in contact with IAN. The purpose of this retrospective cohort study aimed to investigate the neurosensory function after surgical treatment with or without the application of CS.

## Sample and methods

### Sample and study design

The current research project was reviewed and approved by the IRB at the Chonbuk National University before the project began. This study was a retrospective case-control one consisting of patients who underwent mandibular cystic extraction. Radiologic and surgical records were reviewed for the patients who underwent mandibular cyst extraction for 5 years (2014–2018) at the Chonbuk National University Hospital in South Korea. For the preoperative cone beam computed tomography (CBCT) examination, patients who had cystic lesions with the inferior alveolar nerve canal (IAC) invasion were selected. The patients with normal sensation were included, while those who complained about abnormal sensory functions before the enucleation were excluded. Patients complaining of abnormal sensory function after the surgery were subjected to follow-up examinations of between 10 and 66 months.

The records of all patients who were treated for OKC and UAM for the 5 years between 2014 and 2018 were reviewed. Based on the pathology reports, all cases of cystic lesions of the mandible were screened for inclusion in the study. However, the patients who were diagnosed with other subtypes of AM, such as solid/multicystic (i.e., intraosseous) and peripheral ameloblastomas, were excluded. Out of a total of 248 patients with a cystic lesion in mandible, 77 cases with IAC invasion, as shown by CBCT, were selected for further analysis. The analysis included scrutiny of intraoperative findings as documented in the surgical records and the histological diagnosis from incisional biopsies and final enucleation specimens. Oral pathologists reviewed histological sections of all 77 cystic cases. The patients were divided into two groups according to the use (non-use) of CS.

### Surgical procedure

During the 5 years (2014–2018), the treatment for the patients with OKC and UAM in the Department of Oral and Maxillofacial Surgery, the Chonbuk National University Hospital, was consisted of the followings: a biopsy-confirmed diagnosis, enucleation of the lesion and application of CS to the bony cavity, and long-term clinical and radiological follow-up. In performing enucleation of the lesion, the teeth directly related to the periphery of the cyst were extracted before proceeding with the enucleation. If IAN was exposed during the enucleation procedure, the cyst was carefully stripped from the nerve. The bony cavity was then examined for any remaining cystic tissue. If a residue was found, it was removed. CS was applied to the bony cavity for 3 min using cotton applicators or soaked ribbon gauze. However, the use of CS was minimized as much as possible when the mandibular nerve was visible in the bony cavity. This procedure was followed by copious irrigation with normal saline, and clinical and radiological follow-up was carried out.

### Data analyses

Data analyses were performed using SPSS version 25 (SPSS, Inc., Chicago, IL). Descriptive statistics of the sample and the comparison of means and the frequencies between the CS group and non-CS groups were performed by using one-way ANOVA and chi-square tests to assess the relationship between the use of CS and sensory function before the multivariate logistic regression analysis. The dependent variable for the logistic analysis was the subjective numeric rating scale (NRS), and the patient expressed the degree of his or her sensory function in numbers. A value of 10 indicates a high-level sensory function and 0 for no sensation at all. A total of 77 patients were divided into two groups: one group that indicated their sensory scale of between 8 and 10, showing somewhat normal sensory function, and the other group which indicates below 8, showing non-normal sensory function. Therefore, we considered that a patient’s sensory function was almost normal when NRS was eight or above.

On the other hand, the independent variables for the logistic regression analysis included sex, age in years, follow-up period, invasion size of the lesion, and the use of CS. The invasion size had three groups according to the degree of cyst invasion of IAC: less than one third (1), between one third and two thirds (2), and over two thirds of the circumference of the canal (3). Given the nature of the dependent variable, binary logistic regression was used and the invasion size was treated as an interval measure for logistic regression analysis, ranging from 1 to 3.

## Results

### Descriptive statistics

Table 1 shows the descriptive statistics. First of all, among a total of 77 patients in this study, 57 (74%) and 20 of them (26%) were males and females, respectively. Next, among a total of 77 patients, a postoperative biopsy revealed OKC for 14 and UAM for five patients. Out of a total of 77 patients, CS was used for a total of 19 (24.7%) patients who were diagnosed with either OKC or UAM. The postoperative biopsy also revealed dentigerous cysts (DC) for thirty patients (39%) and radicular cysts (RC) for twenty-eight patients (36%). Fifty-seven patients (74%) indicated that their sensory function, NRS, at the end of the follow-up period was between 8 and 10 (normal) and 20 (26%) of them expressed below scale 8 (not normal).

**Table 1:** Descriptive statistics

Variables	Attributes			
Categorical variables				
Sex	Males: 57 (74%) Females: 20 (26%)			
CS use	CS use: 19 (24.7%) No use: 58 (75.3%)			
Sensory recovery	Normal (NRS = 8–10): 57 (74%) Not normal (NRS = below 8): 20 (26%)			
Diagnosis	OKC: 14 (18.2%), UAM: 5 (6.5%), DC: 30 (39%), RC: 28 (36.4%)			
Interval variables	Minimum	Maximum	Mean	Std. deviation
Age	11	76	39.56	14.42
Follow-up period in months	10	66	36.96	13.91
Invasion size	1	3	1.91	0.83
NRS right after cyst enucleation				
Total (77)	02	10	7.94	2.58
CS group (19)	02	10	6.68	3.25
Non-CS group (58)	02	10	8.34	2.20
NRS at the end of the follow-up period				
Total (77)	02	10	8.61	2.14
CS group (19)	02	10	8.00	2.86
Non-CS group (58)	03	10	8.81	1.84

Note: *OKC* odontogenic keratocyst, *UAM* unicystic ameloblastoma, *DC* dentigerous cyst, *RC* radicular cyst, *CS* Carnoy’s solution, *NRS* numeric rating scale

The mean age of the patients was 39.56 and the mean follow-up period was 36.96 months. The average NRS right after the surgery was 7.94, and the one at the end of the follow-up period was 8.61. Therefore, on average, there was only a 0.67 improvement of NRS at the end of the follow-up from the one right after the surgery. Finally, the mean invasion size was 1.91.

### General description of sensory function

Out of 14 OKC patients, the cyst recurred only for one patient (7.14%) during the 5-year follow-up period. For two patients with nevoid basal cell carcinoma (NBCC), multiple OKCs were found in various areas of the jaw, but no lesion recurred. Eight patients complained of abnormal sensory function (NRS = below 8) immediately after the treatment with CS. However, four of them reported that they recovered their sensory function and expressed their sensory function of NRS at between 8 and 10 during the follow-up period. On the other hand, the other four OKC patients who were treated with CS (28.57%) still expressed abnormal sensory function during the follow-up period.

Among five patients in the UAM group, four patients complained of abnormal sensory function immediately after the treatment with CS. Three of them had almost recovered sensory function with NRS of from eight to ten during the follow-up period, and one of them still complained of discomfort due to sensory dysfunction at the end of the follow-up period. Therefore, five out of 19 patients (26%) who were treated with CS developed permanent abnormality of sensory function. Twenty out of 58 patients (35%) with DC and RC whose nerve exposures were confirmed and who underwent cyst enucleation without the application of CS complained of abnormal sensory function immediately after surgery. Fifteen of these 58 patients (26%) still complained of sensory dysfunction (NRS below 8) during the follow-up.

The average NRS immediately after the surgery for the patients without CS treatment was 8.34, and the one for those with CS was 6.68 and the one-way ANOVA test indicated a significant difference in NRS between these two groups (*p* < 0.05). However, the NRS at the end of the follow-up period for the patients without CS was 8.81 and the one for those with CS was 8.00. One-way ANOVA test suggested no significant difference in NRS at the end of the follow-up period between these groups. Likewise, as discussed earlier, the incidence of sensory dysfunction (NRS below 8) was 26% in the CS use group and 26% in the non-CS use group, and the chi-square test indicated no significant difference in sensory function at the end of the follow-up period between the two groups.

Table 2 shows the relationship among the use of CS, the invasion size of the lesion, and sensory function recovery. Chi-square tests were inappropriate because the counts in many cells were smaller than five. Instead, Fisher’s exact test was used [10]. Within non-CS use group, the non-normal sensory function is likely to occur when the invasion size is large (Fisher’s exact test value = 30.2, *p* < 0.001). The percentages of patients who indicated normal sensory recovery were 100%, 85%, and 28% for invasion size one, two, and three, respectively. However, within the CS use group, the invasion size is not related to sensory function recovery (Fisher’s exact test = 1.4, not significant). The bivariate relation analysis did not control other variables that may also be related to the recovery of normal sensory function. Therefore, a clear conclusion must come from the logistic regression analysis.

**Table 2:** The use of CS and invasion size of lesion and patients’ sensory recovery during the follow-up period

CS use	Invasion size	Non-normal sensory recovery	Normal sensory recovery	Total	Fisher’s exact test
No	1	0 (0%)	27 (100%)	27 (100%)	30.2, *p* < 0.001
	2	2 (15%)	11 (85%)	13 (100%)	
	3	13 (72%)	5 (28%)	18 (100%)	
	Subtotal	15 (26%)	43 (74%)	58 (100%)	
Yes	1	0 (0%)	3 (100%)	3 (100%)	1.4, not significant
	2	3 (27%)	8 (73%)	11 (100%)	
	3	2 (40%)	3 (60%)	5 (100%)	
	Subtotal	5 (26%)	14 (74%)	19 (100%)	
Total		20 (26%)	57 (74%)	77 (100%)	

### Binary logistic regression results

Table 3 shows the results of the binary logistic regression analysis. Only the invasion size was significantly and negatively related to the recovery of normal sensory function at a 0.001 level. One unit increase of the invasion size was associated with a 92% decrease in the odds of recovering normal sensory function. However, the use of CS along with other variables such as sex, age, and the follow-up period was not significantly associated with the recovery of normal sensory function. Given Nagelkerke *R*^2^, the variables in the regression model explained 51.7% of the variation of the normalness of sensory function at the end of the follow-up period.

**Table 3:** Binary logistic regression analysis of normalness of sensory function (*N* = 77)

Independent variables	Regression coefficient (S.E. in parentheses)	Odds ratio (95% confidence interval in parentheses)
Sex	0.160 (0.773)	1.173 (0.258–5.341)
Age	− 0.025 (0.026)	0.975 (0.926–1.027)
The follow-up period	0.007 (0.023)	1.007 (0.962–1.054)
Invasion size	− 2.488 (0.599)^***^	0.083 (0.026–0.269)
CS use	− 0.079 (0.792)	0.924 (0.196–4.359)
− 2 log-likelihood	54.766	
Nagelkerke *R*^2^	0.517	

Note: ***p < 0.001

## Discussion

Odontogenic keratocyst (OKC) and ameloblastoma (AM) are commonly developed in the posterior mandibular region and have a high recurrence rate [11]. OKC is one of the dental cysts, and it is known to have an aggressive tendency and high recurrence rate because a patient does not feel pain before it is discovered by chance. There is a two-third chance that it is more frequent in the mandible and occurs about three times more frequently in the posterior molar than in the premolar, mostly in the bone, and 50% of OKCs occur concerning the impacted teeth. Residues in the epithelial lining of keratocyst have been reported to have intrinsic growth potential [12].

Next, AM is a benign but locally aggressive odontogenic tumor that develops in the enamel organs of the dental tissue, the ameloblast. This lesion is the second most frequent oral tumor after OKC. AM is most commonly developed in the mandible (85%) and is rare in the maxilla and elsewhere (15%) [13]. Seventy percent of them occur in the posterior and ramus area in the mandible. Most AMs are derived from epithelial rests in the bone tissue but sometimes occur directly from the surface or epithelial rests of the dental lamina located on the outer surface of the bone. The recurrence rate of AM after relatively conservative treatments is high, ranging from 50 to 90%, except for the case of extensive resection of the tumor [2].

Unicystic ameloblastoma (UAM) is one of the three clinical variants of AM. UAM has become established as a distinct clinicopathological entity on the general basis of its unicystic radiographic appearance, histologic findings, association with an unerupted tooth, and occurrence in the mandible of younger patients. UAM represents the clinical and radiological characteristics of the common cyst that occurs in the jaw, but histologically represents the typical ameloblastoma epithelium that surrounds the cyst. UAMs are considered to be less deformed and less aggressive than OKC, with or without lumen or wall tumor proliferation. UAM’s recurrence rate after conservative surgical treatment is lower than that of its conventional counterpart [14]. In this study, only UAM was studied among different variants of AM.

Because UAM is quite similar to a dentigerous cyst, the clinical, radiological, and biological behavior of these two types of cyst groups have been researched. In addition, the recurrence of OKC and UAMs can occur over a long period, so long-term postoperative follow-up is essential for proper management [15, 16]. OKC and UAM proliferate slowly but have a similar tendency to be locally invasive and prone to recurrence [1, 17, 18]. Therefore, the surgical procedure of these lesions requires a complete removal or degeneration of the daughter’s sac or remaining cells around the lesion to reduce the recurrence rate. Among the surgical methods to reduce the recurrence rate of lesions, resections that sacrifice extensive bones around the lesions have the lowest recurrence rates but have the disadvantage that the surgery itself leads to large morbidity. Therefore, enucleation with Carnoy’s solution (CS) has been suggested as an alternative surgical method that contributes to a low recurrence rate [3, 12].

In spite of such a possible advantage of using CS, there has been concern that CS can degenerate normal tissue and cause nerve damage. To evaluate the impact of CS on nerve damage, we evaluated sensory function by dividing patients into the control group with cyst enucleation only and the experimental group with CS treatment as well as the enucleation. Even if chemical cauterization is applied to the exposed IAN for 19 patients in this study, the majority of patients did not experience sensory dysfunction. In addition, the recovery of sensory function was achieved in a relatively short period so that they feel comfortable in daily life.

Overall, the cyst enucleation did not cause a serious sensory dysfunction right after the surgery for groups, both with and without CS treatment. On average, the NRS for all 77 patients right after the surgery was 7.94 and only a little improvement of 0.67 at the end of the follow-up period (8.61) was observed. Additionally, it appeared that a large invasion size of the legion is related to an increased odd of sensory dysfunction for the non-CS use group, but the invasion size is not associated with sensory dysfunction for the CS use group. However, a substantial variation existed for individual patients. For example, NRS right after the enucleation for two patients who were treated with CS was two and the NRS for them was still two at the end of the follow-up period. Therefore, a further in-depth research is required to examine why some patients sustain severe sensory damage right after the enucleation with CS treatment and their sensory functions did not recover at the end of the follow-up period.

A few limitations of the present research deserve a brief discussion. First, the sample size was small and the number of experimental (19) and control groups (58) was not balanced. Second, the patients in the experimental and control groups had different types of lesions and the lesions may be larger and more invasive to IAC among the OKC and UAM patients with CS treatment than among those with DC and RC. Therefore, future researchers need to randomly divide the patients with OKC and UAM into the experimental and control groups to test the effect of the use of CS on the sensory dysfunction. Third, the sensory evaluation may not be accurate, depending on the subjective feeling of a patient. Finally, the follow-up period is somewhat short and not consistent across patients.

## Conclusions

Although it may be premature to generalize the findings of this study due to the small sample size, it may be advisable to apply CS to reduce recurrence and morbidity without much concern of sensory dysfunction. However, future researchers need to continue studying this topic by using a large sample, diverse populations, and in-depth analysis.

## Data Availability

All data generated or analyzed during this study are included in this published article.

## Appendix

**Table 4:** Raw data

No.	Diagnosis	Sex	Age	Name initial	Follow-up period (month)	Invasion size	Preoperative NRS	Right after operation NRS	Follow-up NRS
1	OKC	F	26	OSB	32	2	10	2	2
2	OKC	F	25	KSE	55	3	10	7	9
3	OKC	F	16	KJH	30	2	10	10	10
4	OKC	F	13	HSB	22	2	10	3	10
5	OKC	M	62	BBJ	19	1	10	10	10
6	OKC	M	62	YSI	16	2	10	10	10
7	OKC	M	54	SHI	23	1	10	10	10
8	OKC	M	46	LTK	33	2	10	5	5
9	OKC	M	46	SYH	56	2	10	10	10
10	OKC	M	45	CCH	54	3	10	2	2
11	OKC	M	38	LSH	33	2	10	7	9
12	OKC	M	37	OJW	24	2	10	2	5
13	OKC	M	20	BJW	19	1	10	8	10
14	OKC	M	11	LSH	51	2	10	10	10
15	UAM	F	23	KJE	63	2	10	3	10
16	UAM	F	18	LYJ	19	3	10	7	8
17	UAM	F	17	JJS	20	2	10	10	10
18	UAM	M	53	KK	57	3	10	3	4
19	UAM	M	17	MJB	22	3	10	8	8
20	DC	F	57	JSN	42	2	10	10	10
21	DC	F	55	JSD	37	2	10	9	9
22	DC	F	46	CJ	51	2	10	8	8
23	DC	F	30	LJA	24	1	10	10	10
24	DC	M	76	PKB	23	2	10	10	10
25	DC	M	59	BSW	26	3	10	3	3
26	DC	M	58	LNJ	60	1	10	10	10
27	DC	M	56	CYH	66	3	10	9	10
28	DC	M	56	HBK	39	2	10	10	10
29	DC	M	55	BWH	34	1	10	10	10
30	DC	M	54	LYJ	36	1	10	9	10
31	DC	M	53	OJK	52	1	10	10	10
32	DC	M	53	KKH	32	1	10	8	8
33	DC	M	52	KHK	21	2	10	10	10
34	DC	M	50	LJE	24	1	10	10	10
35	DC	M	50	KDH	19	2	10	10	10
36	DC	M	44	KDY	11	3	10	5	7
37	DC	M	40	JSK	34	1	10	10	10
38	DC	M	39	JIC	49	3	10	5	7
39	DC	M	39	KSW	51	3	10	7	9
40	DC	M	36	KYH	29	1	10	10	10
41	DC	M	35	KH	51	2	10	10	10
42	DC	M	34	SWM	44	3	10	9	9
43	DC	M	33	JYH	41	3	10	9	9
44	DC	M	30	KYM	38	2	10	10	10
45	DC	M	29	JJH	33	2	10	10	10
46	DC	M	28	SH	31	1	10	9	9
47	DC	M	28	OSH	35	1	10	10	10
48	DC	M	23	LJH	28	1	10	10	10
49	DC	M	17	KHD	59	1	10	8	9
50	RC	F	61	PYH	35	1	10	10	10
51	RC	F	46	OMJ	19	3	10	7	7
52	RC	F	43	JYH	37	1	10	10	10
53	RC	F	40	LSA	41	1	10	10	10
54	RC	F	35	OHL	58	3	10	6	6
55	RC	F	34	KHK	65	3	10	7	7
56	RC	F	32	LHJ	51	3	10	5	5
57	RC	F	31	CHS	43	1	10	10	10
58	RC	F	23	PDS	10	3	10	7	7
59	RC	M	59	LMS	47	1	10	10	10
60	RC	M	59	CBS	29	1	10	8	10
61	RC	M	52	KHK	27	1	10	10	10
62	RC	M	51	PYK	55	3	10	7	7
63	RC	M	50	HSK	23	1	10	10	10
64	RC	M	49	NSK	39	3	10	5	5
65	RC	M	49	LYC	34	3	10	6	7
66	RC	M	43	PSM	27	1	10	9	10
67	RC	M	42	LDJ	49	1	10	10	10
68	RC	M	41	OYM	51	1	10	10	10
69	RC	M	39	PSH	40	3	10	3	3
70	RC	M	37	LJY	58	2	10	5	7
71	RC	M	36	KJW	38	2	10	4	6
72	RC	M	32	PSS	37	1	10	10	10
73	RC	M	32	WSD	22	1	10	10	10
74	RC	M	30	CHJ	48	3	10	8	10
75	RC	M	21	SYK	31	2	10	2	10
76	RC	M	19	JDI	24	1	10	10	10
77	RC	M	16	LSB	40	3	10	7	7

## Abbreviations

AM: Ameloblastoma
CBCT: Cone beam computed tomography
CS: Carnoy’s solution
DC: Dentigerous cyst
IAC: Inferior alveolar nerve canal
IAN: Inferior alveolar nerve
NBCC: Nevoid basal cell carcinoma
NRS: Numeric rating scale
OKC: Odontogenic keratocyst
RC: Radicular cyst
UAM: Unicystic ameloblastoma
